# Hereditary multiple exostoses and schizophrenia

**DOI:** 10.4103/0971-6866.44107

**Published:** 2008

**Authors:** Germán Gòmez-Bernal

**Affiliations:** Department of UME, C.R.P San Juan de Dios (UME), Avenida de Zaragoza No 10, 44001, Teruel. Spain

**Keywords:** Schizophrenia, multiple exostoses, genetics

## Abstract

I report a case of a patient who suffered schizophrenia and multiple exostoses and argue the possible role of EXT gene and nearly chromosomal loci in further genetic investigations related to schizophrenia.

In 1989 Aizenberg *et al.*, presented a case history of two members of a family with multiple exostoses, ventricular brain enlargement and psychosis.[1]

## Case Report

**Figure 1:**
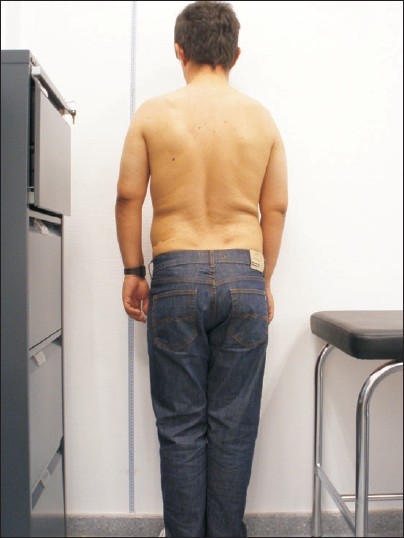
Short height of the patient. Notice three scars in the back secondary to osteochondroma removal

We report the case of a 36-year-old male, who was admitted in October 2007 and diagnosed with DSM-IV schizophrenia. He had personal and family history of multiple exostoses [Figure 1], but no family history of psychosis. Psychiatric manifestations of our case are similar to Aizenberg's cases: Age of onset (around 20), labile and incongruent effect, lack of insight, marked paranoid delusions, no perceptual disturbances, lack of response to neuroleptic treatment, and poor social and laboral adjustment. The results of general blood chemistries were within normal limits. Brain magnetic resonance imaging showed a little ventricular enlargement.

After the patient's psychotic behavior began, several trials were performed with high doses of various neuroleptic and mood stabilizer drugs (Haloperidol, Risperidone, Ziprasidone, Divalproex, Lithium…). Augmentation and combination strategies and electroconvulsive therapy were also tried with poor response, and several psychiatric hospitalizations were required. Finally, a slow and partial response to the combination of Olanzapine 20 mg/day, Haloperidol 20 mg/day, Lamotrigine 300 mg/day, clonazepam 2 mg/day and Zuclopenthixol 40 mg/day was obtained.

The patient suffered from multiple osteochondromas near the ends of long bones (mainly in his legs), which needed surgical orthopedic interventions when he reached late adolescence.

## Discussion

Multiple exostoses is a genetically heterogeneous disease with at least three chromosomal loci: EXT1 (Chromosome 8), EXT2 (Chromosome 11), EXT 3 (Chromosome 19). An autosomal dominant pattern of transmission has been described, although approximately 20% of reported cases have no family history of the disease.[2] Exostoses are rarely present at birth, but gradually arise and increase in size with age with a wide spectrum of clinical presentation: from only radiologically distinguishable signs, to different skeletal deformities that remain minor physical anomalies of schizophrenics.[3]

Genetic transmission, phenotype-genotype correlation, variable spectrum of clinical manifestations and disease courses are very similar in both multiple exostoses and schizophrenia. All these shared characteristics and the cases reported, lead us to believe in the important role of EXT genes and nearly chromosomal loci, in further genetic investigations related to schizophrenia.

## References

[1] Aizenberg D, Blumensohn R, Shalev A, Munitz H (1989). Multiple exostoses, brain ventricular enlargement and schizophrenia. Psychiatr J Univ Ott.

[2] Alvarez C, Tredwell S, De Vera M, Hayden M (2006). The genotype-phenotype correlation of hereditary multiple exostoses. Clin Genet.

[3] Alvarez C, De Vera M, Heslip TR, Casey B (2007). Evaluation of the anatomic burden of patients with hereditary multiple exostoses. Clin Orthop Relat Res.

